# Occurrence, Distribution and Risk Assessment of Mercury in Multimedia of Soil-Dust-Plants in Shanghai, China

**DOI:** 10.3390/ijerph16173028

**Published:** 2019-08-21

**Authors:** Yanzhuo Liu, Shanshan Song, Chunjuan Bi, Junli Zhao, Di Xi, Ziqi Su

**Affiliations:** 1 Key Laboratory of Geographic Information Science (Ministry of Education), East China Normal University, Shanghai 200241, China; 2 School of Geographic Sciences, East China Normal University, Shanghai 200241, China; 3 Institute of Eco-Chongming (IEC), East China Normal University, Shanghai 202150, China

**Keywords:** mercury, soil, road dust, leaves, foliar dust, LUR, health risk

## Abstract

The urban environment is a complex ecosystem influenced by strong human disturbances in multi-environmental media, so it is necessary to analyze urban environmental pollutants through the comprehensive analysis of different media. Soil, road dust, foliar dust, and camphor leaves from 32 sample sites in Shanghai were collected for the analysis of mercury contamination in soil–road dust–leaves–foliar dust systems. Mercury concentrations in surface soils in Shanghai were the highest, followed by road dust, foliar dust, and leaves, successively. The spatial distribution of mercury in the four environmental media presented different distribution patterns. Except for the significant correlation between mercury concentrations in road dust and mercury concentrations in leaves (r = 0.56, *p* < 0.001), there was no significant correlation between the other groups in the four media. Besides this, there was no significant correlation between mercury concentrations and land types. The LUR (Land use regression) model was used to assess the impact of urbanization factors on mercury distribution in the environment. The results showed that soil mercury was affected by factories and residential areas. Foliar dust mercury was affected by road density and power plants. Leaf mercury was affected by power plants and road dust mercury was affected by public service areas. The highest average HI (Hazard index) value of mercury in Shanghai was found in road dust, followed by surface soil and foliar dust. The HI values for children were much higher than those for adults. However, the HI values of mercury exposure in all sampling sites were less than one, suggesting a lower health risk level. The microscopic mechanism of mercury in different environmental media was suggested to be studied further in order to learn the quantitative effects of urbanization factors on mercury concentrations.

## 1. Introduction

Mercury (Hg), one of the most toxic heavy metals in the environment [1,2], is often present in ecosystems in Hg^0^, Hg^+^, and Hg^2+^ [3,4]. Elemental mercury tends to form colorless and odorless mercury vapors, which can remain in the atmosphere for long periods and can be transported over long distances [5,6]. Compared with elemental mercury, ionic mercury sinks more rapidly from the atmosphere to surface ecosystems because of its shorter retention period [5,7]. In environmental media, mercury can be complexed with or sequestered by a variety of inorganic or organic ligands, as well be reduced or methylated to form stable pollutants [6,8]. Those pollutants are generally bio-enriched in the environmental media (water, dust, and soil, etc.) and can be enlarged through the food chain, eventually entering the human body and posing a threat to human health [9]. With the development of industry, mercury pollution has been paid more attention [10].

Urban soil with high spatio-temporal variability has been greatly influenced by multiple human disturbances for a long time. Mercury is mainly enriched in surface soils, which has a wide range of natural and anthropogenic sources and can be continuously migrated to other environmental media through processes such as surface runoff and re-suspension to the air [11,12]. Urban surface dust is mainly solid particulate matter that was deposited on the urban impervious surface originating from urban atmospheric deposition, soil, traffic, construction dust, and combustion [13,14], which is a complex secondary primary pollutant that is easily enriched with heavy metals [15]. Plants can generally absorb the ionic state and dissolve gaseous mercury in soil through root [16], meanwhile they can absorb the gaseous elemental mercury (Hg^0^) in the atmosphere through the stomatal respiration of leaves [17]. Generally speaking, mercury concentration in plant leaves is much higher than that in roots [18]. Foliar dust can absorb the bivalent active gaseous mercury and particulate mercury in the atmosphere, becoming an important medium for mercury enrichment [19].

The contamination sources of heavy metals can be identified by various methods, such as principal component analysis, multiple linear regression (PCA-MLR), positive matrix factorization (PMF), etc. [20,21]. However, there are relatively few studies focusing on social factors affecting mercury emissions and distribution, including land use, population migration, industrial layout, and energy structure. In recent years, land use regression (LUR) models have been used to extract high-resolution urbanization features (e.g., land use, socio-economic, traffic data, etc.) around sampling sites to assess the spatial distribution characteristics and influencing factors of some environmental pollutants [22,23]. The LUR model is still not used to simulate the spatial distribution of mercury pollution in different media in cities. Therefore, it is necessary to further analyze the key factors in urbanization characteristics affecting mercury pollution in different environmental media.

Thus far, numerous studies focused on the concentration, source, and health risks of various heavy metals in the soil-dust system in urban ecosystems [24,25,26,27]. Studies have shown that mercury in road dust is more concentrated than that in soil and mercury contamination in both media is strongly influenced by human factors, especially industrial activities [24,25]. Urban residents will have certain health risks through exposure to contaminated soil and dust [26,27]. The health risks of mercury exposure to children were generally higher than those to adults [27], and the risks of exposure to road dust were higher for individuals than those in soil [26]. Meanwhile, some attention have been paid to the occurrence characteristics and health risks of heavy metals in green plants or vegetables in cities [28,29]. As a good detector of urban atmosphere pollution, mercury concentration in plant leaves and foliar dust was closely related to plant species [28,29]. However, research on mercury distribution characteristics in leaves and foliar dust in combination with the soil–dust system in urban environments is not sufficient. The aims of this study are (i) to analyze mercury concentrations in surface soil, road dust, foliar dust, and tree leaves in Shanghai; (ii) to explore the spatial distribution characteristics of mercury in four media; (iii) to evaluate the influencing factors of mercury in different media using the LUR model; and (iv) to assess the potential health risks of mercury in four environmental media. The result can be used to improve the efficiency of mercury pollution control in multi-environmental media during urbanization and provides a theoretical basis for controlling the risk of mercury exposure to urban residents.

## 2. Materials and Methods

### 2.1. Study Area

Shanghai is located in the middle of the curved coastline of Eastern China and is the Easternmost part of the Yangtze River Delta (30°40′–31°53′ N, 120°51′–122°12′ E). With an area of 6340.5 km^2^, it is one of the largest cities in the world and also the center of finance, trade, and shipping in China. According to the statistic in 2017, the GDP (Gross domestic product) in shanghai reached over 3000 billion yuan, in contrast with 780 billion yuan in 1990. However, the rapid economic growth is at the expense of large-scale energy consumption. Energy consumption increased from 32 million tons of standard coal in 1990 to 119 million tons in 2017, with an average annual growth rate of 10.07% [30]. With the rapid development of urbanization, heavy-metal concentrations in various media in the city has increased [22,31,32]. As a dominant species in the Yangtze River Delta, camphor trees (*Cinnamomum camphora*) are often used as a test organism for pollutants [33].

**Figure 1:**
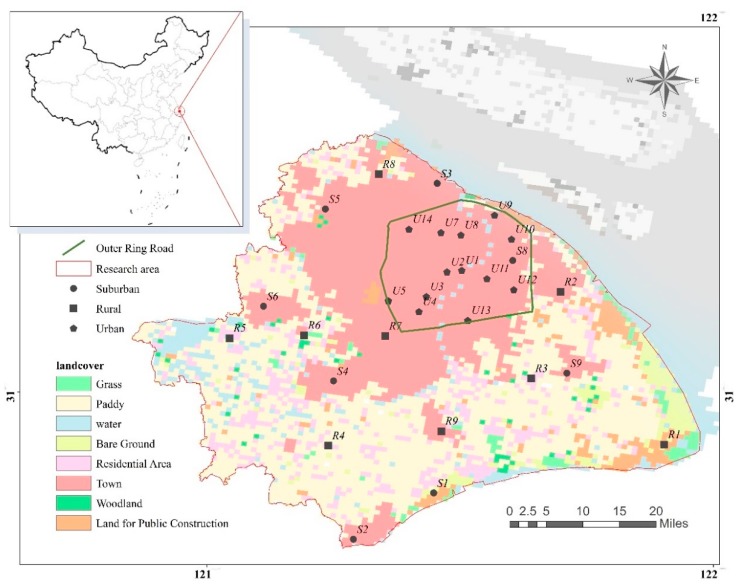
Schematic diagram of the sampling sites and their land use type.

According to the characteristics of urban functional area, 32 sampling sites were selected, including 14 urban sites (U1–U14) near intensive traffic network or concentrated residential and commercial areas, 9 rural sites (R1–R9) in typical agricultural areas or low and middle-income residential areas, and 9 industrial sites in suburban areas (S1–S9) (Figure 1). The samples were collected on 19 December 2018 when it was cloudy and about 12 °C. The dry period before rain was 5 days.

### 2.2. Sample Collection

The 0–5 cm surface soil samples were taken using a stainless–steel shovel. The road dust samples were collected from the impervious roadside surface using a hairbrush. Both the soil and the road dust samples were formed into composite samples from more than five subsamples, respectively. High-twig shears were used to collect one branch of the camphor tree leaves at least 3 m above the ground from four different directions, and at least 5 trees were collected at each sampling site. The leaves were carefully removed and then mixed into a plastic bag as a composite sample. A total of 128 samples of four environmental media out of 32 sample sites were collected. All the samples were brought back to the laboratory in 2 h and then stored at −20 °C until further analysis.

### 2.3. Sample Preparation and Analysis

Soil and road dust samples were dried at 40 °C, then ground and passed through a 100-mesh nylon screen. Leaves were washed with ultrapure water and shaken three times with an ultrasonic oscillator for at least 4 min each time. The extracting solution was filtered through a 0.45 µm aqueous phase filter (drying at 105 °C for 24 h before use) and the filter was weighed after lyophilization.

The solid phase samples were determined according to the reported methods [34]. 0.25 g soil or road dust sample was placed in a 10 mL colorimetric tube and then 5 mL freshly prepared aqua regia and 5 mL pure water was added into the tube. After being heated in a 95 °C water bath for 1 h, the digestion solution volume was fixed to 10 mL. Take the supernatant for analysis of mercury concentration. Foliar dust on the filter was directly digested using the above method.

After foliar dust was removed, Camphor tree leaves were lyophilized and then cut with ceramic scissors. 0.5 g leaf samples were accurately weighed into a 50 mL colorimetric tube and then 40 mL concentrated nitric acid was added into the tube. After being heated in a 70 °C water bath and placed overnight, the digestion solution volume was fixed to 50 mL. Take the supernatant for analysis of mercury concentration.

The concentrations of mercury in digestion solutions were measured by AFS 9230 double-channel atomic fluorescence spectrometer (Beijing Jitian Co., Ltd., Beijing, China). The measurement conditions of the instrument are shown in Table S1. The 5% HCl (V/V) carrier fluid and 1% KBH_4_ + 0.5% KOH (m/V) reductant were prepared with ultrapure water (18.2 MΩ·cm) on the day of use. A mercury standard stock solution (1000 mg/L, China Institute of Metrology) was diluted in 5% nitric acid (V/V) + 0.05% potassium dichromate (m/V) solution to prepare a mercury working solution (1.0 µg/L).

Blanks, spikes, and parallel samples were simultaneously analyzed for quality assurance and quality control. The relative standard deviation of each set of parallel samples was controlled within 10%. Chinese national standard materials GSS-2, GSS-3, and GSV-3 were used to calculate the recovery rate. The spiked recovery rate of each batch of samples was 64.7%–79.2%.

### 2.4. Land Use Regression Model (LUR)

Based on the main influencing factors of mercury pollution, four sets of predictors (land use types, traffic variables, industrial sources, and population densities) were selected to assess their impact on spatial variation of mercury concentrations in urban environmental media (Table 1).

The LUR model was established on the basis of existing researches [35,36]. Firstly, a correlation analysis was performed between mercury concentrations in different media for each sample and each predictor. The correlated (*p* < 0.05) variable was taken into further calculation. Secondly, for each variable category (i.e., land use type, traffic variable, industrial source, and population in our study), variables that have the highest correlation coefficient were picked to make a correlation with other variables. To avoid collinearity, all the highly correlated (r > 0.6) variables were removed and other variables were taken in their corresponding category. Finally, stepwise Linear regression analyses were used with all the extracted variables to get the LUR model. Variables added to the final LUR model should increase the adjusted R^2^ by more than 1% and had statistical significance (*p* < 0.05). Cross-validation (LOOCV) was performed to calculate the correlation coefficient (R) and root mean square error (MSE) between the measured and predicted concentrations of all samples to assess the uncertainty of the model. In the model, R^2^, adjusted R^2^ and MSE were used to assess the model’s performance.

### 2.5. Health Risk Assessment Model

Health risk assessment was calculated for children and adults, respectively, mainly because of their behavior and physiology differences. Mercury exposure to residents mainly occurred through the following pathways: (1) Direct ingestion of particles, (2) inhalation of dust particles, and (3) dermal contact absorption. According to the USEPA Health Risk Handbook [37], the exposure doses via these pathways can be calculated as:

(1) $$ADD_{ing}=c\times \frac{IR_{ing}\times CF\times EF\times ED}{BW\times AT}$$

(2) $$ADD_{inh}=c\times \frac{IR_{inh}\times EF\times ED}{PEF\times BW\times AT}$$

(3) $$ADD_{dermal}=c\times \frac{SA\times SL\times ABS\times CF\times EF\times ED}{BW\times AT}$$

where *ADD_ing_* is the direct ingestion dose (mg/(kg·day)); *ADD_inh_* is the inhalation dose through mouth and nose (mg/(kg·day)); *ADD_dermal_* is the dermal absorption dose (mg/(kg·day)); *c* is mercury concentration in different environmental media (mg/kg); and the explanations of other parameters are listed in Table 2.

The non-carcinogenic risk was characterized by Hazard quotient (HQ) and calculated by the following Equations [42,43]:

(4) HQ=ADD/RfD

(5) $$HI=\sum HQ_{i}$$

where *ADD* is the exposure dose calculated above (mg/(kg·day)); *RfD* is the reference dose through a certain pathway, which is also the maximum permissible risk (mg/(kg·day)); and *i* stands for pathways. The *RfD* for each exposure route was listed in Table S2. Hazard index (*HI*) is the sum of *HQ* of each exposure route. *HI* < 1 indicates that there is no adverse health risk and *HI* ≥ 1 indicates the possible health risk [43].

## 3. Results and Discussion

### 3.1. Mercury Concentrations in Different Environmental Media

The descriptive statistics of total mercury concentrations in surface soils, road dust, foliar dust, and camphor tree leaves in Shanghai are summarized in Table S3. The average total mercury concentration in surface soils is 0.36 mg/kg, which is 3.6 times of the soil background value of 0.1 mg/kg and is about twice that of 10 years ago [42]. Compared with other cities (Table 3), the average mercury concentration in soils in Shanghai is higher than that of the contrast cities, except for San Luis Potosí, México, which has experienced a long-term mining process [43]. However, the maximum value of soil mercury in Shanghai is moderate compared with other cities and is lower than that of most other Chinese cities.

The average mercury concentration in road dust in Shanghai is 0.596 mg/kg, which is higher than that in soils, suggesting the stronger mercury enrichment ability in dust. This is probably due to the fine particle size of dust because the larger specific surface area makes it easier to concentrate heavy metals on the surface [44]. In comparison with other cities (Table 3), mercury concentration in road dust is also high. The average concentration in Shanghai is higher than that of Baoji City, which has developed the mining industry for a long time. The relatively high mercury concentration in road dust in Shanghai is largely due to the improper disposal of municipal waste caused by high-speed urbanization process [45] and the multi-channel emissions of environmental pollutants caused by the long-term accumulation of the chemical industry [46].

Unlike the study results by Yin et al. in this area [47], mercury concentrations in foliar dust in this study is lower than those in road dust (Table 4). Mercury concentrations in foliar dust were related to the characteristics of the leaves [48] and its concentrations in the airborne particles in a short period of time [49]. The relatively low concentrations of mercury in foliar dust might be due to the relatively weak adsorption of camphor tree and the superior air quality during the sampling period. Comparison results between different plant species in the temperate or subtropical region showed that mercury concentrations in leaves and foliar dust had a significant difference (Table 4). The concentrations of heavy metals in foliar dust of different plants showed a significant correlation with the amount of road dust on the leaves [50], while the later was affected by the micromorphology and contact angle of leaves [51]. Actually, different plant leaves have different absorption capacities for mercury due to their different fiber, protein, and the amount of -SH contained in the molecular structure [52]. Therefore, the higher mercury concentrations of the leaves of Shanghai camphor tree compared with other regions might be due to its better adsorption capacity or higher mercury concentrations in the Shanghai atmosphere.

### 3.2. Spatial Variation of Mercury in Different Environment Media

**Figure 2:**
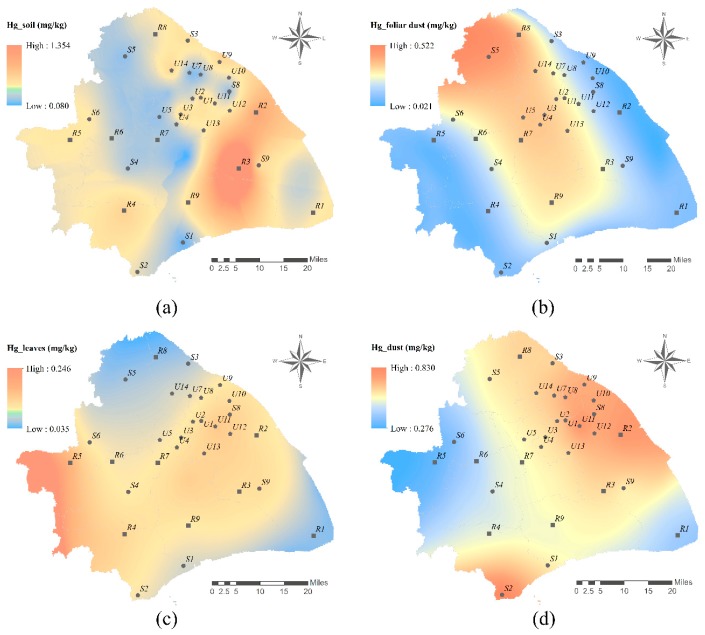
Interpolation map of mercury concentrations in four media in Shanghai, (**a**) soil, (**b**) foliar dust, (**c**) camphor tree leaves, and (**d**) dust. R: rural area (R1–R9); S: suburban industrial area (S1–S9); U: urban area (U1–U14).

Pearson correlation analysis results showed that there was no significant correlation between mercury concentrations in soils and other environmental media (Table S4). As shown in Figure 2a, soil mercury concentrations showed uneven spatial distribution with a high-value block in the East-central area. The maximum mercury concentration was in an ancient town (R3) with a history of over 800 years, which was prosperous for the salt making industry. In the process of salt making, lots of insoluble chemical components in brine (such as calcium, magnesium, and mercury) were discharged [76]. As time goes by, the mercury on the land surface would transfer into soil slowly with the erosion of wind and rain. It can be inferred that the salt industry makes the high soil mercury concentration there. Similar results were also found in the Zhongba site, the largest salt production center of the Eastern Sichuan province in history [77]. Besides, relative high mercury concentrations were measured in samples to the Northwest of the ancient town, which correspond to the city center with high-density traffic and high intensity human activity. Similarly, a high level of soil mercury relative to the human activities has also been found in other cities [55,59]. The Southwest suburbs also show high mercury concentration, while the middle part of Shanghai showed a low level of mercury. The distribution of mercury concentrations in the two areas was consistent with their soil organic matter (SOM) concentrations [78], in accordance with the reported results that mercury was likely to be bound with SOM in surface soil and thus the concentrations of SOM can be used to estimate the distribution of mercury storage [79].

Foliage dust mercury concentrations showed no significant correlation with both soil mercury and road dust mercury concentrations (Table S4). The spatial distribution of foliage dust mercury concentrations in Shanghai showed a U-pattern distribution characteristic with a high level of mercury in the Northwest area and decreasing gradually to the Southeast (Figure 2b). The high-level area belongs to the urban and industrial area of Shanghai with plenty of exhaust from vehicles emission and factories [80], which might be the main reason for the increasing mercury concentrations in foliar dust. In addition, the particles in foliar dust were found to be too small to settle down and thus could be transported by wind for a long distance [81]. Studies found that pollutants transported from the North and West areas to Shanghai contributed to PM (particulate matter) air pollution events in the city [82]. Since mercury concentrations in foliar dust were closely related to mercury concentrations in the atmosphere [49], the U-pattern distribution characteristics were probably caused by the prevailing wind direction of Northwest (Figure S1) in the winter of Shanghai [83].

Mercury concentrations in road dust showed significant correlation with those in tree leaves (r = 0.56, *p* < 0.001) (Table S4), which is consistent with the reported findings [47,64]. The spatial distribution of mercury in leaf tissues and road dust showed similar trends to a large degree (Figure 2c, d). The source of mercury in the leaves is mainly gaseous mercury in the atmosphere [84], and the mercury contained in the dust particles mainly exists in the vapor state adsorbed on the surface [85], resulting in a certain consistency of mercury concentrations in the two media. The hot spots of road dust mercury concentrations in Figure 2d is highly correlated with two significant industrial areas in Shanghai, i.e., Baoshan industrial area in Northwest Shanghai and Jinshan industrial zone in Southwest Shanghai. The energy structure of the type of production (power generation, coking, petrochemical, etc.) in these industrial areas were both dominated by coal and coke, which was an important source of soot and dust [86]. According to a previous study, emission from Baosteel Group Corporation shared almost 80 percent of the total dust emission in the Baoshan district in Shanghai from 2010 to 2015 [87]. Since road dust was comprised of particles from both the local area and nearby areas, mercury concentrations in road dust showed a decreasing trend from the two industrial zones to the surrounding areas. Different from road dust, mercury in leaf tissue showed high concentrations in the Western area of Shanghai. It might be because the road dust can be more obviously affected by human activities such as cleaning habits or foot disturbance, so mercury concentrations in road dust can be more variable than those in leaf tissue.

**Figure 3:**
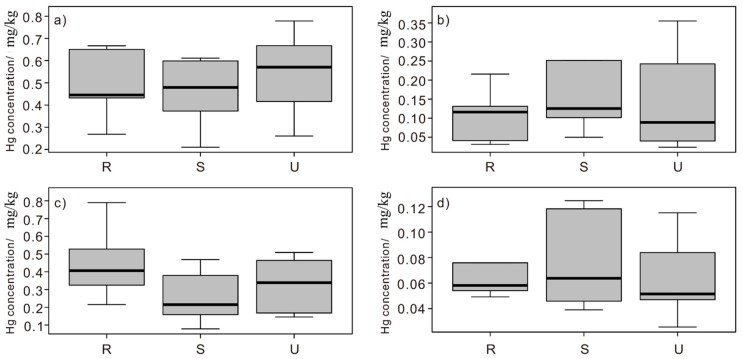
Comparison of mercury concentrations in different land-use types of four environmental media, (**a**) road dust, (**b**) foliar dust, (**c**) soil, and (**d**) camphor tree leaves. R: rural area (R1–R9); S: suburban industrial area (S1–S9); U: urban area (U1–U14). The solid lines within boxes show the median values of each group. The upper and lower boundary of the boxes indicates the 25th and 75th percentiles. Horizontal lines represent the maximum and minimum values.

Three land-use types (rural area, suburban industrial area, and urban area) were identified in Shanghai in our study. The Scheffe test was used to test whether there was significant difference between two land use types. The results showed that there was no significant difference of mercury concentration between different land use types in four environmental media (Figure 3), suggesting the complexity of mercury sources in the environment media. Our result was almost consistent with the findings in Beijing [53], except for the classic garden, a land use type influenced by mercury in red pigment used in historical buildings. Different to Beijing, Shanghai is a modern city with an escalating rate of urbanization and active land use change situation. According to a recent study, the resident area dramatically increased, transforming from another type of land like arable land, industrial area, etc. [88]. Since soil and leaves both reflect the long-term accumulation of heavy metal, the influence of land-use type on mercury concentration might be weakened [89,90]. Besides, due to the launch of a new town project, an increasing number of suburban areas in Shanghai developed into a complex industrial and residential area [91]. In this way, the distance between the two areas is declining, which means that they may share the same pollution source. Regarding the foliar dust, the mercury concentration can be influenced by the PM particles, which are easily transported by wind [92]. Thus, the point source pollution can affect the environmental media in a large degree area that might include various land use types.

### 3.3. Influencing Factor Analysis Using LUR Model

The LUR model was used to analyze the influencing factors of mercury distribution in foliar dust, surface soils, road dust, and camphor tree leaves (Table 5). Adjusted R^2^ indicates the model performance. R^2^ greater than 0.7, range of 0.5–0.7, and less than 0.5 means good performance, acceptable performance and considerably poor, respectively [35,36]. The correlation coefficient and mean square error (MSE) between predicted and measured values from the LOOCV analysis reflects the prediction ability of a model. In general, a higher correlation coefficient and less MSE means better accuracy of the model.

**Figure 4:**
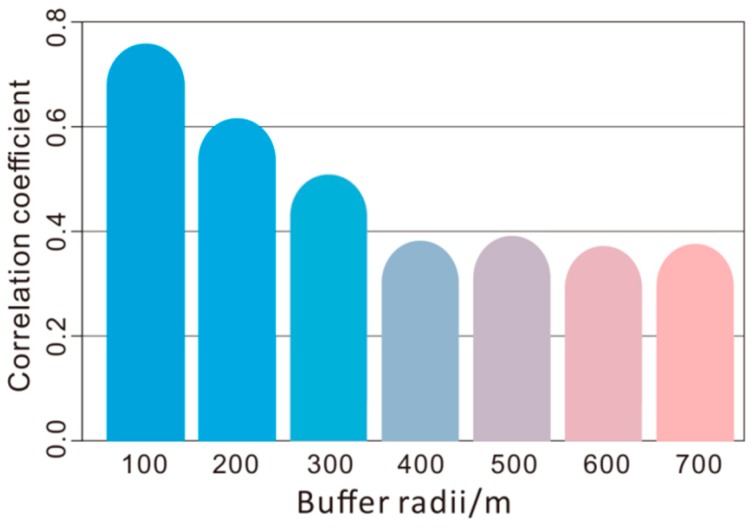
Correlation coefficients between road density and mercury concentrations in foliar dust for different buffer radii.

As shown in Table 5, the LUR model performed well for foliar dust (adjusted R^2^ > 0.8). The built LUR model revealed that mercury concentrations in foliar dust were associated with road density within 100 m buffer area and the number of power plants within 2000 m buffer area, indicating that vehicles and power industries were potential sources of mercury in foliar dust. Coal-fired power plants had been regarded as the largest source type in the inventory for mercury in many countries [93]. Approximately 40% of mercury released by power plants existed as oxidized mercury and particle-bound mercury, which could deposit within 80 to 800 km of emission source and thus increased mercury concentration around there [93]. In Shanghai, the elevated particulate mercury concentration was also detected around two power plants [94]. Except for firing coal, gasoline vehicles were another chief emission source of both gaseous elemental mercury and particle-bound mercury [95]. The correlation coefficients between mercury concentrations in foliar dust and road density declined significantly as the buffer radius extended (r = −0.896, *p* < 0.01) (Figure 4). It has been found that particulate matter pollution in the traffic zoning displayed a decreasing trend with the distance away from the road [96]. Through the above relationship, it can be inferred that vehicle emission was a major source of mercury in foliar dust.

Similar with foliar dust, the number of power plants was also included in the LUR model of leaves (Table 5). However, the model performance was not as well as the foliar dust (adjusted R^2^ < 0.5), which was mainly because of the different sources of mercury in leaf tissues. Plant leaves absorb heavy metals mainly via the cuticle and the stomatal pores. The former channel mostly absorbs particle-bound heavy metals, while the latter usually absorbs mercury in the gaseous phase [97]. Additionally, Birbaum et al. found that fine particles were more likely to be incorporated into the leaves, whereas large particles could only be captured as the foliar dust [98]. Given these considerations, mercury in leaves has different sources from that in foliar dust, although they are both influenced by power plant. Specifically, leaves absorb mercury vapor rather than particulate mercury. The gaseous elemental mercury, emitted by power plants and transported in a long distance, can be detained in the air for months to a year and be absorbed by leaves. In this way, inter-continental transport of mercury is possible. Travnikov et al. (2002) revealed that approximately 46% of annual mercury deposition was from the outer source of other continents by modeling [99]. A large amount of outer source might contribute to the poor performance of the LUR model because it just took local influence factors into consideration. It is also in accordance with the result that no relationship was shown between mercury concentrations in foliar dusts and leaves.

Urban soils have been highly disturbed by frequent human activities. For mercury in surface soils, the residential area within 200 m buffer area and the factory number within 1000 m buffer area were introduced into the LUR model, although it had a poor performance. Factories in our LUR model include sewage treatment plants, waste incineration plants, livestock farms, and heavy metal products companies. Studies showed that the possible anthropogenic inputs of mercury to urban land were the application of fertilizers, the fly ash from coal boilers, disposal of residential solid waste, disposal of municipal incinerator ash, sewage sludge, the production of non-ferrous metals, and other sources [94,100,101]. Therefore, it is reasonable for mercury concentrations in soils increasing with the number of factories. However, in our study, most soil samples were collected from the green belts due to the accessibility. Nowadays, most of the green belts were fertilized with sewage sludge due to its high contents of organic matter and low cost [102]. While there was a low level of mercury in sludge from 12 sewage treatment plants [103], the accumulation effect of heavy metals in green belts might also change the spatial distribution characteristic of soil mercury in Shanghai, which also reduced the accuracy of our LUR model.

As for mercury in road dust, although public service area was included in the LUR model, the bad performance (adjusted R^2^ < 0.5) showed that mercury concentrations in road dust could not be explained mainly by one factor. Except for the automobile exhaust particles and fly ash from industrial activities, Kennedy and Gadd (2003) found that wear particles of tyres, brake linings, and bitumen also contributed to mercury concentrations in road dust [104]. Shi (2008) also suggested that soil-weathering particles were another significant source [105]. Besides, compared with soils or foliar dust, road dust could also be easily influenced by anthropogenic activities, such as cleaning, treading, and so on. All these factors led to the complexity and multiplicity of mercury sources in road dust, which decreased the precision of the model.

### 3.4. Health Risk Assessment

The non-carcinogenic health risk for adults and children through three exposure pathways was calculated based on Equations (1)–(3), and then the result was summarized in Table 6. In order to better understand the influence of human activities in Shanghai city, 32 sample sites were divided into three land use types in this study (urban area, industrial area, and rural area).

Compared with adults, children had more health risks of mercury. More specifically, the average HI values for children were approximately nine times those for adults, which was similar to other studies [60,106]. Children have different behavior and physical characteristics from adults that can increase their possibility of exposure threat from mercury. For example, children have a higher respiration rate per unit body weight as well as an increased absorption ability of gastrointestinal tract, and repetitive hand/finger sucking is another pathway for children to ingest more dust [107,108]. For both children and adults, ingestion of soil or dust particles was the main pathway, followed by dermal contact absorption, while the risk caused by air inhalation was nearly negligible. Similar ranking was also reported in other studies [62].

Regardless of receptor age, the HI values in the rural area were the highest among all the land types, followed by the industrial area. In the rural area, HI values for either adults or children decreased in the order of road dust > soils > foliar dust. In the industrial area, road dust was also the most significant mercury source for both children and adults. One possible explanation is the dense traffic volume resulting from transporting products. In the urban area, people also faced the greatest health risk from exposure to mercury in road dust, whilst they took similar but less health risks from mercury in soils and foliar dust. Overall, road dust contributed the most to the potential health risks of mercury in the whole study areas compared to soils and foliar dust.

Considering all the possible mercury exposure pathways and sources, the HI values for all land-use types were within the safe level of one, indicating little adverse health risk for residents in Shanghai. However, except for the environmental media we studied, mercury could be introduced to human bodies through other ways such as food, water, etc. [109]. Since most of the heavy metals cannot be excreted from human bodies, they can be accumulated in the body for a long time. Hence, the potential non-carcinogenic health risk of mercury cannot be negligible.

## 4. Conclusions

Mercury can transfer in the soil-dust-leaves-foliar dust system and influence each other in these four environmental media. Mercury concentrations in the four environmental media were at the midstream level compared with other cities in the world and showed the highest values in road dust. Due to the inconsistency of mercury sources, there was no significant correlation between mercury concentrations in different environmental media except for mercury concentrations in camphor tree leaves and road dust (r = 0.56, *p* < 0.001). Additionally, due to the rapid change of land types caused by the urbanization process and the intertwined complex urban pattern of various land types, no significant correlation existed between mercury concentrations and land types in the four environmental media. Urbanization factors such as road density, number of power plants, and area of public service areas all had an effect on mercury concentrations using the LUR model. Vehicle emission was a major source of mercury in foliar dust. However, the low fitting accuracy in the LUR model indicated the diversity and complexity of sources of mercury contamination in the urban environmental media. The health risk assessment results showed that residents were less exposed to mercury contamination. In general, children were more exposed to mercury than adults, and the exposure risk to mercury in residential areas was the highest. Mercury in dust poses the highest health risks to humans among the four environmental media. Considering the complexity of the exposed environment for urban residents, the pollution characteristics of mercury in other environmental media should also be paid more attention.

## Figures and Tables

**Table 1:** Potential predictor variables influencing mercury distribution.

Variable Type	Subcategory	Buffer Radius (m)	Data Sources
Land use type	Residential area	500, 1000, 1500, 2000, 2500, 3000	Shanghai Land Use Classification Map (2014)
	Business district		
	Public service area		
	Industrial area		
	Traffic facility area		
	Other		
Traffic variable	Distance to city expressway	50, 100, 200, 300, 400, 500, 600, 700, 800, 900, 1000	OpenStreetMap data (2017)
	Distance to the highway		
	Distance to national highway		
	Road network density		
Industrial source	Number of power plants	2000,5000,10,000, 15,000	Yang et al. [22]
	Number of national pollutant control factory		
Population	Population density	1000, 2000, 5000	Shanghai Statistical Year Book [30]

**Table 2:** Parameters for health risk model of mercury.

Parameter	Definition	Unit	Value		Reference
			Children	Adult	
*SA*	Surface area exposed	cm^2^	852.5	1610	Wang et al., 2008 [38]
*AT*	Average exposure time	day	365 × *ED*	365 × *ED*	USEPA, 2004 [39]
*EF*	Exposure frequency	day/a	180	180	Ferreira-Baptista and De Miguel, 2005 [40]
*IR_inh_*	Inhalation rate	m^3^/day	7.6	20	Van den Berg, 1994 [41]
*IR_ing_*	Ingestion rate	mg/day	200	100	USEPA, 2001 [37]
*ED*	Exposure duration	a	6	24	
*CF*	Conversion factor	kg/mg	10^−6^	10^−6^	
*BW*	Average body weight	kg	15	70	
*PEF*	Particle emission factor	mg^3^/kg	1.36 × 10^9^	1.36 × 10^9^	
*SL*	Skin adherence factor	mg^3^/kg	0.2	0.07	
ABS	Dermal absorption factor		0.001	0.001	

**Table 3:** Mean, minimum, and maximum concentrations of mercury (mg/kg) in soil and road dust in some cities.

City or Country	Hg in Soil (mg/kg)			
	Mean	Min	Max	Reference
Shanghai, China	0.361	0.078	1.362	This study
Beijing, China	0.3	0.022	9.4	Chen et al. (2010) [53]
Guangzhou, China	0.334	0.025	3.32	Chen et al. (2012) [54]
Shenzhen, China	0.09	0.017	0.02	Chen et al. (2012) [54]
Wuhan, China	0.207	0.024	2.844	Fang et al. (2011) [55]
Slovenia	0.106	0.012	5.293	Gosar et al. (2016) [56]
San Luis Potosí, México	0.45		2.34	Perez-Vazquez et al. (2015) [57]
South Carolina, U.S. (resident)	0.024		0.22	Liu et al. (2010) [58]
Athens, Greece	0.166	0.01	1.08	Kelepertzis et al. (2015) [59]
	**Hg in road dust (mg/kg)**			
Shanghai, China	0.596	0.210	2.184	This study
Beijing, China	0.16	0.04	0.78	Men et al. (2018) [60]
Nanjing, China	0.12	0.05	0.34	Hu et al. (2011) [61]
Huainan, China	0.16	0.02	0.56	Zheng et al. (2015) [62]
Nanning, China	0.338	0.045	0.804	Lin et al. (2018) [63]
Xiamen, China	0.28	0.034	1.4	Ying et al. (2009) [64]
Baoji, China	1.11	0.48	2.32	Lu et al. (2009) [25]
Brno, Czech Republic		0.03	2.67	Coufalík et al. (2014) [65]
Luanda, Angola	0.13	0.03	0.57	Ferreira-Baptista et al. (2005) [40]
Kavala, Greece	0.13		3.3	Christoforidis et al. (2009) [66]
Tijuana, México	0.1		0.3	Qui~nonez-Plaza et al. (2017) [67]

**Table 4:** Mean, minimum, and maximum concentrations of mercury (mg/kg) in foliar dust and plant leaves in some cities.

City or Country	Plant Species	Hg in Leaves (mg/kg)			
		Mean	Min	Max	Reference
Shanghai, China	Camphor tree	0.088	0.026	0.453	This study
Zhuzhou, China	Cinnamomum camphora	13.64	2.6	22.9	Chen et al. (2002) [68]
Harbin, China	woody tree, shrub	0.113	0.004	0.772	Mu et al. (2004) [69]
Minnesota, U.S.	Tamarack	0.037			Laacouri et al. (2013) [70]
Slovakia	Achillea millefolium L.(herb)		0.019	0.055	Dombaiová et al. (2005) [71]
	Corylus avellana L., Carpinus betulus L., Salix fragilis L. and Quercus polycarpa Schur. (broadleaves)		0.022	0.052	
	Picea abies (L.) H. Karst. (needles)		0.014	0.053	
Jódar, Spain	Olea Europea, L.(olive-tree)	160.6	46	453	López-berdonces et al. (2014) [72]
		**Hg in Foliar Dust (mg/kg)**			
Shanghai, China	Camphor tree	0.259	0.024	2.260	This study
Inner Mongolia, China (coal-mining area)	Leymus chinensis	0.251	0.05	0.42	Li et al. (2016) [73]
Yerevan, U.S.	white elm; Chinese elm; Persian walnut; oriental plane tree; common lilac; white poplar; white mulberry tree	0.57	0.03	2.37	Maghakyan et al. (2017) [74]
Vanadzor, U.S.	Ulmus parvifolia L.; Juglans regia L.; Fraxinus ex- celsior; Acer platanoides L.; Populus alba L.; Populus nigra L	0.57	0.027	3.295	Sahakyan et al. (2018) [75]

**Table 5:** Land use regression model for mercury in foliar dust, soil, road dust, and leave.

Target Compound	Model	Adjusted R^2^	LOOCV	
			R	MSE (mg/kg)
Foliar dust	Y = 0.127 + 427.953 × road_density_100 + 0.418 × power_plant_number_2000	0.810	−0.06	0.43
Soil	Lg(Y) = −1.290 + 0.057 × lg(residential_area_200) − 0.259 × lg(factory_number_10000)	0.303	0.47	0.27
Road dust	Y = 0.362 + 0.000000204 × public_service_area_1500	0.133	−0.09	0.15
Leaves	Lg(Y) = −2.693 + 1.253 × log(power_plant_2000)	0.138	−0.91	0.36

Factory_number means the sum number of sewage treatment plants, waste incineration plants, livestock farms, and heavy metal products companies. Public_service_area means the sum area of municipal utilities, stadiums, and parks. LOOCV means Leave One Out Cross Validation.

**Table 6:** Hazard quotient (*HQ*) for different exposure pathways in soils, dust and foliar dust in different land-use types.

Children		Urban Area	Rural Area	Industrial Area
Soil	*HQ_ing_*	6.1810 × 10^−3^	1.2110 × 10^−2^	6.3510 × 10^−3^
	*HQ_inh_*	6.0510 × 10^−7^	1.1910 × 10^−6^	6.2110 × 10^−7^
	*HQ_dermal_*	7.5310 × 10^−5^	1.4810 × 10^−4^	7.7310 × 10^−5^
	*HI*	6.2610 × 10^−3^	1.2310 × 10^−2^	6.4310 × 10^−3^
Road dust	*HQ_ing_*	1.0710 × 10^−2^	1.6510 × 10^−2^	1.3210 × 10^−2^
	*HQ_inh_*	1.0510 × 10^−6^	1.6210 × 10^−6^	1.2910 × 10^−6^
	*HQ_dermal_*	1.3110 × 10^−4^	2.0110 × 10^−4^	1.6110 × 10^−4^
	*HI*	1.0910 × 10^−2^	1.6710 × 10^−2^	1.3410 × 10^−2^
Foliar dust	*HQ_ing_*	6.1610 × 10^−3^	3.0410 × 10^−3^	7.5810 × 10^−3^
	*HQ_inh_*	6.0310 × 10^−7^	2.9710 × 10^−7^	7.4210 × 10^−7^
	*HQ_dermal_*	7.5010 × 10^−5^	3.7010 × 10^−5^	9.2310 × 10^−5^
	*HI*	6.2410 × 10^−3^	3.0810 × 10^−3^	7.6810 × 10^−3^
All media	*HI*	2.3410 × 10^−2^	3.2110 × 10^−2^	2.7510 × 10^−2^
**Adults**				
soil	*HQ_ing_*	6.6210 × 10^−4^	1.3010 × 10^−3^	6.8010 × 10^−4^
	*HQ_inh_*	3.4110 × 10^−7^	6.6910 × 10^−7^	3.5010 × 10^−7^
	*HQ_dermal_*	1.0710 × 10^−5^	2.0910 × 10^−5^	1.1010 × 10^−5^
	*HI*	6.7310 × 10^−4^	1.3210 × 10^−3^	6.9210 × 10^−4^
Road dust	*HQ_ing_*	1.1510 × 10^−3^	1.7710 × 10^−3^	1.4110 × 10^−3^
	*HQ_inh_*	5.9310 × 10^−7^	9.1110 × 10^−7^	7.2810 × 10^−7^
	*HQ_dermal_*	1.8510 × 10^−5^	2.8510 × 10^−5^	2.2810 × 10^−5^
	*HI*	1.1710 × 10^−3^	1.8010 × 10^−3^	1.4410 × 10^−3^
Foliar dust	*HQ_ing_*	6.6010 × 10^−4^	3.2610 × 10^−4^	8.1210 × 10^−4^
	*HQ_inh_*	3.4010 × 10^−7^	1.6810 × 10^−7^	4.1810 × 10^−7^
	*HQ_dermal_*	1.0610 × 10^−5^	5.2410 × 10^−6^	1.3110 × 10^−5^
	*HI*	6.7110 × 10^−4^	3.3110 × 10^−4^	8.2610 × 10^−4^
All media	*HI*	2.5110 × 10^−3^	3.4510 × 10^−3^	2.9610 × 10^−3^

## Supplementary Materials

The following are available online at https://www.mdpi.com/1660-4601/16/17/3028/s1, Table S1: Determination parameters of AFS 9230 atomic fluorescence photometer. Table S2: Reference dose (*RfD*) for each exposure pathways. Table S3: Descriptive statistics of mercury concentrations (mg/kg) in soil, dust, foliar dust and camphor tree leaves in Shanghai. Table S4. Pearson correlation coefficient (r) of mercury concentrations in soils, dust, foliar dust and camphor tree leaves in Shanghai. Figure S1. Shanghai winter (December - February) wind rose.
