# Supplementary material for: Occurrence, Distribution and Risk Assessment of Mercury in Multimedia of Soil-Dust-Plants in Shanghai, China
Source: Int J Environ Res Public Health. 2019 Aug 21;16(17):3028. doi: 10.3390/ijerph16173028 (PMC6747141; doi:10.3390/ijerph16173028)
Supplement: Supplementary file 1 [file ijerph-16-03028-s001.pdf]

Table S1. Determination parameters of AFS 9230 atomic fluorescence photometer.

Instrument condition	Parameters	Instrument condition	Parameters
Negative high pressure	285 V	Reading time	12 s
Lamp current	30 mA	Delay time	0.5 s
Atomizer height	12 mm	Measurement method	Std. Curve
Atomizer temperature	200 ℃	Reading method	Peak Area
Carrier gas flow rate	300 mL/min	Effective measurement times	2
Shielding gas flow	800 mL/min		

Table S2. Reference dose (*RfD*) for each exposure pathways.

Pathways	Reference dose/ mg/(kg×day)
Direct ingestion	3.00E-04
Inhalation of particles	8.57E-05
Dermal absorption	2.10E-05

Table S3. Descriptive statistics of mercury concentrations (mg/kg) in soils, dust, foliar dust and camphor tree leaves in Shanghai.

Descriptive	Soil	Dust	Foliar dust	Leaves
Mean	0.361	0.596	0.259	0.088
Medium	0.3364	0.5019	0.1132	0.0564
Std. Deviation	0.240	0.366	0.482	0.083
CV %	66.4	61.5	185.8	93.8
Minimum	0.078	0.210	0.024	0.026
Maximum	1.362	2.184	2.260	0.453

Table S4. Pearson correlation coefficient (r) of mercury concentrations in soils, dust, foliar dust and camphor tree leaves in Shanghai. (*: Significantly at 99.9% confidence level)

	Soil	Road dust	Foliar dust	Leaves
Soil	1			
Road dust	0.0512	1		
Foliar dust	-0.2335	-0.0559	1	
Leaves	-0.0093	0.5648*	0.0612	1

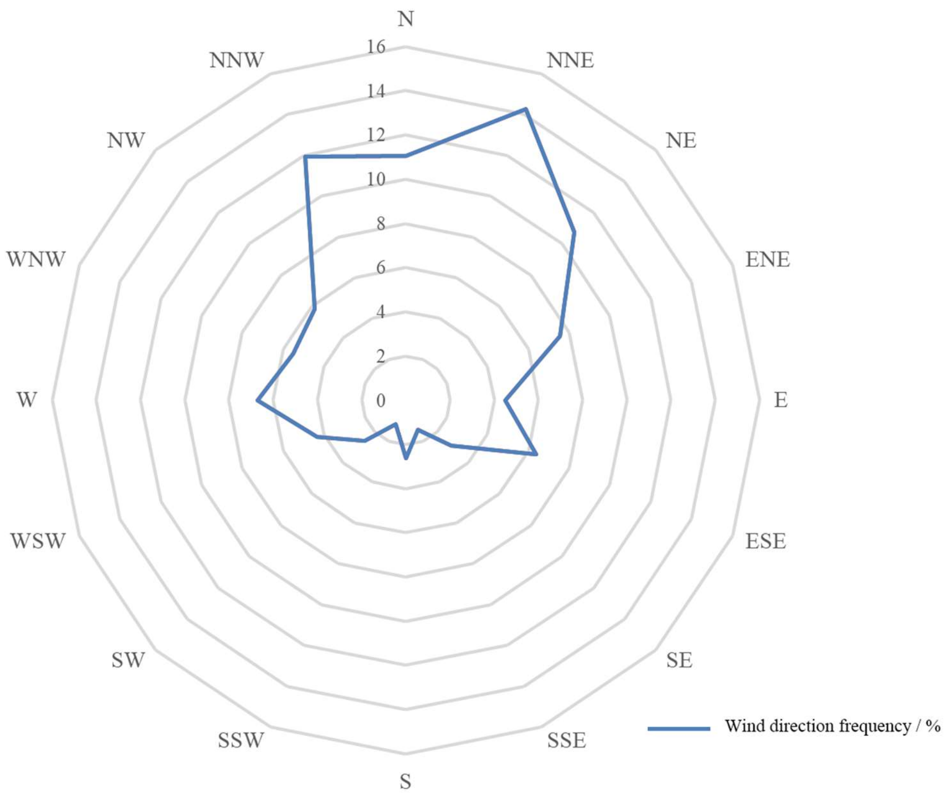

Figure S1. Shanghai winter (December - February) wind rose. The meteorological data are derived from the measured meteorological data of Shanghai from 1971 to 2003 [1].

References

1. Meteorological Information Center of China Meteorological Administration. Special data set for China's building thermal environment analysis. *China Building Industry Press* **2005**. (in Chinese)
